# The Effect of Liver Transplantation on Anti‐Glycaemic Agents in Patients With Pre‐Existing Diabetes Mellitus: A Population‐Based Cohort Study

**DOI:** 10.1111/1753-0407.70088

**Published:** 2025-07-15

**Authors:** Amy Coulden, Christina Antza, Orighomisan Awala, Nihit Shah, Leelavathy Kandaswamy, Ananda Nahar, Angela Phillips, Sneha Upadhyaya, Ayesha Asif, Zara Khan, Feaz Babwah, Matthew Armstrong, Samiul Mostaf, Wasim Hanif

**Affiliations:** ^1^ University Hospitals Birmingham NHSFT Birmingham UK; ^2^ The Department of Metabolism and Systems Science, College of Medicine and Health University of Birmingham Birmingham UK

**Keywords:** diabetes mellitus, glycaemic control, insulin resistance, liver transplantation

## Abstract

**Background:**

Anecdotally, patients with diabetes mellitus after undertaking a liver transplant have reported improved glycaemic control and reduced insulin requirements, compared to other organ transplants where glycaemic control often worsens. This study aimed to evaluate possible changes in anti‐glycaemic agent requirements pre‐ and post‐liver transplantation and correlate them with the immunosuppression used and the reason for transplant.

**Methods:**

In this observational, retrospective study, we investigated 258 adults with pre‐existing diabetes mellitus who underwent liver transplant from October 2009 to January 2020 at a tertiary UK center. We compared pre‐ and post‐transplant insulin treatment requirements.

**Results:**

The mean age was 56 years, and the median duration of diabetes was 96 months. From a subgroup of 100 patients (38.8%) using insulin therapy, there was a reduction in insulin requirements from 60.5 ± 44.6 units/day before transplant to 51.1 ± 31.2 units/day at 1 month post‐transplantation (15.5%, *p* = 0.02), 43.4 ± 29.5 units/day at 3 months post‐transplantation (28.2%, *p* < 0.0001) and 33.6 ± 29.7 units/day at 6 months post‐transplantation (44.4%, *p* < 0.0001). There was a significant correlation between the difference in insulin requirement before and 6 months post‐transplant and the tacrolimus dose used as immunosuppressive therapy post‐liver transplant. There was no correlation with the use of other immunosuppressive therapies and change in insulin requirement.

**Conclusions:**

Insulin requirements significantly reduced post‐liver transplant by almost 50%, despite initiation of immunosuppressive therapy. This is one of the first studies showing this effect, highlighting the role of the liver in regulating glucose metabolism, insulin utilization, and insulin resistance. Pooled data from other specialist centers need to be examined.

## Graphical abstract

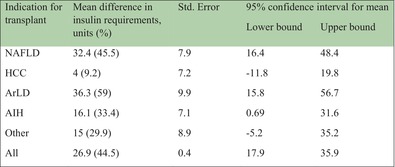

**Summary**

- We found a significant reduction in insulin requirement from 60.5 ± SD 44.6 units/day before transplant to 51.1 ± 31.2 units/day at 1 month post‐transplantation (15.5%, *p* = 0.02), 43.4 ± 29.5 units/day at 3 months post‐transplantation (28.2%, *p* < 0.0001) and 33.6 ± 29.7 units/day at 6 months post‐transplantation (44.4%, *p* < 0.0001).
- The reduction was more marked in patients requiring liver transplant for alcohol‐related liver disease (ArLD) and non‐alcoholic fatty liver disease (NAFLD), although this difference was not statistically significant.
- There was a significant correlation between insulin reduction and the dosage of tacrolimus used as an immunosuppressant post‐transplant, but not between the other types of immunosuppression.
- Severity of liver disease did not correlate with the reduction in insulin requirement.

## 1 Introduction

Diabetes mellitus is common in patients with liver disease requiring liver transplant, largely due to the liver's role in insulin resistance, known as hepatogenous diabetes [1, 2, 3]. Diabetes is associated with poorer outcomes post‐liver transplantation with increased mortality and graft dysfunction [4, 5, 6, 7]. Patients with pre‐existing diabetes mellitus prior to liver transplantation have been shown to have much higher rates of death than patients without diabetes [4, 6, 7], possibly due to hyperglycaemia and poor glycaemic control [4].

Glycaemic control often worsens post‐transplant, with hyperglycaemia occurring in 70% to 90% of all patients (independent of existing diabetes) in the few weeks immediately post‐transplant [8, 9], and an associated rise in fasting blood glucose and glycated hemoglobin (HbA1c) [10]. Factors such as surgical stress, inflammation, and infection and immunosuppressant use have a direct effect on glycaemic control [1, 9]. In many patients without diabetes, this hyperglycaemia can develop into post‐transplant diabetes mellitus (PTDM), which is only considered the diagnosis when the graft function and patient are stable on immunosuppression, without evidence of infection [9].

PTDM is a serious metabolic complication after liver transplantation, with a reported incidence between 14% and 45% one year post‐operation [4]. Use of immunosuppressive agents is the most likely causative factor [8, 11]. Corticosteroids increase hepatic gluconeogenesis, cyclosporin and tacrolimus reduce beta cell density and mass/synthesis, respectively, leading to reduced insulin secretion [12, 13, 14, 15]. This is epitomized by studies showing patients with PTDM going into remission from diabetes (HbA1c < 48 mmol/mol, < 6.5%), once immunosuppression (namely corticosteroids and Calcineurin Inhibitors [CNI] such as tacrolimus) has been reduced and stabilized [10, 11].

In the acute setting, where immunosuppression use is ongoing, worsening glycaemic control in patients with pre‐existing diabetes would therefore be expected, represented by an increase in anti‐glycaemic agent requirements. However, anecdotally this has not been the experience of physicians caring for patients after liver transplantation.

There is a paucity of studies exploring the effect of liver transplantation on glycaemic control in patients with pre‐existing diabetes mellitus. The evidence available is discordant, with reports of no change in insulin requirements [16], an increase in insulin requirements [10] and two case reports describing complete remission of diabetes in patients with long‐standing insulin‐dependent diabetes [3, 17].

There is limited data on the change in anti‐glycaemic treatment use after undertaking liver transplantation, especially total daily insulin requirements. Such information could guide diabetes teams in forming appropriate patient care plans post‐transplant. This study aims to explore the effects of liver transplantation on insulin and oral anti‐glycaemic agents' requirements in patients with pre‐existing diabetes.

## 2 Methods

### 2.1 Study Design

A population‐based, retrospective cohort study, using routinely collected clinical data of patients who underwent liver transplantation between October 2009 and January 2020 at University Hospitals of Birmingham (UHB) NHS Trust, UK. The trust catchment area for liver transplant is vast, accounting for a population of 17 million (one third of the UK liver transplant waiting list).

### 2.2 Data Source

All patients who underwent liver transplantation between October 2009 and January 2020 and had a recorded diagnosis of diabetes mellitus were identified. Relevant clinical data for these patients was then retrospectively extracted from our trusts' Electronic Patient Record (developed internally) consisting of two computer‐based programs: Clinical Portal (portal@UHB, UK) and Prescribing Information Communication System (PICS, UK). A data collection tool were created to collect all variables and information identified. Information was accurately collected by A.C., O.A., A.P., N.S., and P.S. with all patient identifying details kept anonymous.

### 2.3 Study Population, Inclusion and Exclusion Criteria

Patients aged 18 to 80 years old with diabetes mellitus (types 1 and 2), who underwent liver transplantation, were eligible to enter our study. The duration of diabetes mellitus had to be at least 1 year pre‐transplantation. Patients reported as having diabetes mellitus without any record of anti‐glycaemic medications were excluded from the study to prevent misclassification with patients without diabetes. Other exclusion criteria were pre‐existing steroid‐induced diabetes and death within a month of surgery.

Data were collected on demographics, body mass index (BMI), past medical/social history (smoking, alcohol consumption, hypertension and dyslipidaemia), details of diabetes (type, duration and presence of microvascular complications). Laboratory parameters pre‐ and post‐transplant (6 months) were obtained, including HbA1c, random glucose measurements, lipid profile, hemoglobin, and renal function (estimated glomerular filtration rate [eGFR]). Transplant details, including the indication for transplant, severity of disease (Child‐Pugh, UK model for End‐Stage Liver Disease [UKELD] and Model for End‐stage Liver Disease [MELD] scores), type of transplant, and the immunosuppressive regimen (drug and dosage) used upon discharge from hospital were collected.

Our local trust protocol for immunosuppression post liver transplantation is high‐dose corticosteroid immediately post‐surgery, then tapered to stop at 12 weeks, where possible. The exception is autoimmune hepatitis (AIH) where they remain on prednisolone 7.5 mg indefinitely. Tacrolimus and azathioprine are also given and continued, barring no intolerances or side effects, where MMF may be added instead.

Ethical approval was not required for this study.

### 2.4 Outcomes

The primary outcome of the study was to analyze the change in anti‐glycaemic agents' requirement, specifically total daily insulin dose at 1st, 3rd, and 6th month post‐operatively compared to the insulin requirements before liver transplant. This was characterized by total daily insulin dose requirements.

A secondary outcome was the alteration in the use of oral anti‐glycaemic agents over the same time period pre‐ and post‐liver transplant.

### 2.5 Follow Up Period

The follow‐up period began from the date of liver transplant. All data were collected from the day prior to liver transplant and at the 1st, 3rd, and 6th months post‐operatively or until the patient exit date (defined as patient transfer to another GP practice, the final data collection date from their general practice or death). Some data were only collected at pre‐and 6‐months post‐transplant.

### 2.6 Statistical Analysis

Statistical analysis was performed with SPSS 25.0 (SPSS Inc., Chicago, IL) and Microsoft Excel. Continuous variables are presented as mean, standard deviation (SD) and range. Binary and categorical variables are presented as frequencies and percentages.

To compare the differences in insulin titration following liver transplant at different time points within each group separately, repeated measures ANOVA was used, followed by post hoc pairwise comparisons using the Bonferroni adjusted significance (*α* = 0.05). To compare the difference in insulin requirements among different groups, non‐parametric Spearman's Rank‐Order correlation and Kruskal Wallis test were used. Secondary outcomes were estimated using paired Cochran's *Q* test. Paired Student's *t*‐test and chi‐squared analysis were used to assess pre‐ and post‐operative study population characteristics. A *p*‐value < 0.05 was considered statistically significant.

## 3 Results

### 3.1 Study Population Characteristics Pre‐ and Post‐Transplant

The baseline characteristics of our population are summarized in Table 1. The study population consisted of 258 patients, mean aged 55.9 ± 9.6 years old (69% males) who underwent liver transplantation from 2009 to 2020. Most of our population was white Europeans (77.1%) and had a diagnosis of type 2 diabetes mellitus (91.5%). The median duration of diabetes mellitus before transplant was 96 months. The most commonly used diabetes mellitus treatment prior to transplant was insulin therapy (55.9%, *n* = 143), while 54.3% (*n* = 140) were receiving at least one oral anti‐glycaemic agent. Specific data on which agent was not collected. The mean HbA1c was 53.0 ± 7.0 mmol/mol (18.6% ± 3.9%), with an associated mean hemoglobin level of 117.1 ± 20.6 g/dL. Most of the population was free of any microvascular complication of diabetes mellitus (88.1%). The patients were mostly non‐smokers (87.8%), without hypertension (71%) or dyslipidemia (83.4%). The most common indication for liver transplantation was non‐alcoholic fatty liver disease (NAFLD) (33.1%). The severity of liver disease was classified as Child‐Pugh A in 25.5%, Child‐Pugh B in 49.9%, and Child‐Pugh C in 20.3% of patients. The mean UKELD and MELD scores for assessment of severity were 53.8 ± 5.1 and 13.8 ± 5.6, respectively.

**TABLE 1:** Study population characteristics before and 6 months after transplant.

Characteristic		Pre‐transplant	Post‐transplant (6 months)
Demographics			
Age at transplant, years	Mean ± SD	55.9 ± 9.6	
	Range	20–82	
	Total, *n*	257	
Sex, *n* (%)	Male	178 (69.0)	
	Female	80 (31.0)	
Ethnicity, *n* (%)	White European	199 (77.1)	
	African‐Caribbean	1 (0.4)	
	Asian	32 (12.4)	
	Other	2 (0.8)	
	Not specified	24 (9.3)	
Diabetic history and control			
Type of diabetes, *n* (%)	T2DM	236 (91.5)	
	T1DM	22 (8.5)	
Duration of diabetes, months	Median	96	
	Range	12–384	
	Total, *n*	151	
Complications of diabetes, *n* (%)	None	223 (88.1)	
	CKD	12 (4.7)	
	Neuropathy	14 (5.5)	
	Retinopathy	4 (1.6)	
On diabetic medication, *n* (%)	Insulin	143 (55.9)	156 (64.5)
	Oral anti‐glycaemic agents	140 (54.3)	69 (28.0)^e^
	None	0 (0)	38 (15.8)^e^
HbA1c level, mmol/mol(%)	Mean ± SD	53.0 ± 18.6 (7.0 ± 3.9)	48.6 ± 15.9^a^ ^e^ (6.6 ± 3.6)
	Range	20–138 (4–14.8)	17–127 (3.7–13.8)
	Total, *n*	169	169
Random glucose measurement, mmol/L	Mean ± SD	9.7 ± 4.29	12.7 ± 5.6^b^
	Range	1.5–32.7	4–53
	Total, *n*	218	218
Co‐morbidities and associated laboratory values			
Co‐morbidities, *n* (%)	Hypertension	74 (29.0)	93 (36.2)
	Dyslipidemia	40 (16.6)	55 (22.1)
	Non‐smoker	180 (87.8)	224 (96.1)^e^
	Non‐alcohol user	147 (68.7)	230 (98.3)^e^
Hb, g/dL	Mean ± SD	117.1 ± 20.6	114.6 ± 18.1^a^
	Range	48–168	60–157
	Total, *n*	162	162
BMI, kg/m^2^	Mean ± SD	29.5 ± 5.4	27.9 ± 5.3^e^
	Range	17.8–48.6	10.1–47.5
	Total, *n*	234	234
Cholesterol, mmol/L	Mean ± SD	3.7 ± 1.5	4.4 ± 1.2^e^
	Range	1.7–11.2	2.2–7.6
	Total, *n*	66	66
LDL cholesterol, mmol/L	Mean ± SD	1.8 ± 0.5	1.6 ± 0.8
	Range	1.1–2.7	1.0–3.1
	Total, *n*	11	11
Triglycerides, mmol/L	Mean ± SD	1.3 ± 0.84	2.4 ± 1.5^e^
	Range	0.4–6.2	0.6–9.7
	Total, *n*	101	101
eGFR, mL/min/1.73m^2^	Mean ± SD	75.4 ± 18.4	54.6 ± 19.0^e^
	Range	1–90	12–90
	Total, *n*	225	225
Transplant details			
Indication for transplant, *n* (%)	NAFLD	84 (33.1)	
	HCC^c^	39 (15.4)	
	ArLD	70 (27.6)	
	AIH	29 (11.4)	
	Haemochromatosis	5 (2.0)	
	Sarcoidosis	1 (0.4)	
	Other	27 (10.6)	
Type of transplant, *n* (%)	DCD	69 (32.4)	
	DBD	144 (67.6)	
Child Pugh Score, *n* (%)	A	59 (25.5)	
	B	115 (49.8)	
	C	47 (20.3)	
	No cirrhosis	10 (4.3)	
UKELD	Mean ± SD	53.8 ± 5.1	
	Range	42–72	
	Total, *n*	258	
MELD	Mean ± SD	13.8 ± 5.6	
	Range	4–37	
	Total, *n*	244	
Length of hospital stay for liver transplantation, days	Median	11	
	Range	5–134	
	Total, *n*	258	
Post‐transplant immunotherapy regime^d^, *n* (%)	Prograf (Tacrolimus)	252 (97.7)	
	MMF	143 (56.1)	
	Prednisolone	252 (98.4)	
	Azathioprine	106 (41.4)	
Prograf (Tacrolimus) dosage, mg	Mean ± SD	7.1 ± 3	
	Mode	6	
	Range	2–20	
MMF dosage^d^, mg	Mean ± SD	1.8 ± 0.3	
	Mode	2	
	Range	1–2	
Prednisolone dosage^d^, mg	Mean ± SD	19.1 ± 3.4	
	Mode	20	
	Range	5–40	
Azathioprine dosage^d^, mg	Mean ± SD	108.7 ± 18.2	
	Mode	100	
	Range	75–150	

*Note:* Where applicable, where *n* does not equal 258, data was not available.

Abbreviations: AIH: autoimmune hepatitis; ArLD: alcohol‐related liver disease; CKD: chronic kidney disease; DBD: donation after brain death; DCD: donation after circulatory death; HCC: hepatocellular carcinoma; MELD: model for end‐stage liver disease; MMF: mycophenolate mofetil; NAFLD: non‐alcoholic fatty liver disease; SD: standard deviation; UKELD: UK model for end‐stage liver disease.

^a^ 3‐months post‐op.

^b^ 1‐month post‐op.

^c^ As the primary indication for liver transplant.

^d^ Post‐operative regimen on initial discharge from hospital.

^e^ The difference between pre‐op and post‐op is significantly different using either paired Student's *t*‐test or Chi‐squared test.

Upon discharge from hospital after the transplant (median length of stay 11 days), 97% of patients were taking tacrolimus (most frequently used dose: 6 mg), 98.5% were taking prednisolone (most frequently used dose: 20 mg), 56.1% were taking MMF (most frequently used dose: 2 mg) and 41.4% were taking azathioprine as their immunosuppressant therapy post‐transplant (most frequently used dose: 100 mg).

At 6 months post‐transplant (Table 1), the percentage of non‐smokers was statistically higher (96.1%) than before transplant (*p* < 0.005). Higher rates of hypertension (36.2%) and dyslipidemia (22.1%) were observed in the population, although they were not statistically significant. The mean HbA1c level was 48.6 ± 15.9 (6.6% ± 3.6%), showing better control after transplant (*p* < 0.005). The mean hemoglobin level associated with HbA1c did not significantly change (114.6 ± 18.1 g/dL), suggesting recovery of any anemia associated with the operation. There was a significant reduction in renal function from an eGFR of 75.4 ± 18.4 to 54.6 ± 19.0 mL/min/1.73m^2^. There was a non‐significant increase in insulin use to 64.5% of the population, accompanied by a significant reduction in oral anti‐glycaemic agents to 28% (*p* < 0.0001) at 6 months after transplant. Patients no longer requiring any anti‐glycaemic agents significantly increased to 15.8%, *p* < 0.0001.

### 3.2 Impact on Insulin Requirements (Subpopulation Analysis)

The subpopulation descriptive characteristics are summarized in Table 2. Of the 143 patients on insulin prior to liver transplantation (55.9%), 100 (38.8%) were included in the subgroup analysis for insulin reduction. The others were excluded due to lack of data availability at all data points or death. The mean total insulin requirement was 60.5 ± 44.6 units/day pre‐transplant. The requirements reduced compared to pre‐transplant to 51.1 ± 31.2 units/day (15.5% reduction), 43.4 ± 29.5 units/day (28.2% reduction), and 33.6 ± 29.7 units/day (44.5% reduction) at 1‐, 3‐, and 6‐months post‐transplantation, accordingly. Repeated measures ANOVA showed there was a significant difference in mean daily insulin requirements between the time points (*p* < 0.0001). Post hoc pairwise comparisons showed that the greatest mean difference in the reduction of insulin requirements was between the requirements before transplant and those at 6 months post‐transplantation (26.9 ± 0.41 units/day, *p* < 0.0001). Table 3 shows the differences in daily insulin requirements between the four groups (pre‐transplant and 1‐, 3‐ and 6‐months post‐transplant).

**TABLE 2:** Description characteristics for subpopulation patients receiving insulin pre‐transplant.

Characteristic		Pre‐transplant	Post‐transplant (6 months)
Demographics			
Age, years	Mean ± SD	55.8 ± 10.2	
	Range	22–82	
	Total, *n*	99	
Sex, *n* (%)	Male	63 (63)	
	Female	37 (37)	
Diabetic history and control			
Type of diabetes, *n* (%)	T2DM	91 (91)	
	T1DM	9 (9)	
Duration of diabetes, months	Median	120	
	Range	12–384	
	Total, *n*	71	
On diabetic medication, *n* (%)	Insulin	100 (100)	84 (84)^e^
	Oral anti‐glycaemic agents	22 (22)	14 (14.1)
	None	0 (0)	12 (12.1)^e^
HbA1c level, mmol/mol(%)	Mean ± SD	55.7 ± 21.7 (7.2 ± 4.1)	51.7 ± 18.8^a^ (6.9 ± 3.9)
	Range	20–138 (4–14.8)	17–127 (3.7–13.8)
	Total, *n*	72	72
Random glucose measurement, mmol/L	Mean ± SD	9.8 ± 4.9	12.8 ± 6.6^b^
	Range	1.5–32.7	4–53
	Total, *n*	88	88
Co‐morbidities and associated laboratory values			
Hb, g/dL	Mean ± SD	113.1 ± 19.8	114.8 ± 17.7^a^
	Range	69–155	78–157
	Total, *n*	67	67
BMI, kg/m^2^	Mean ± SD	29.3 ± 5.4	27.1 ± 5.5^e^
	Range	17.8–48.6	10.1–42.0
	Total, *n*	92	92
Cholesterol, mmol/L	Mean ± SD	4.0 ± 2.0	4.5 ± 1.1
	Range	1.7–11.2	2.4–7.6
	Total, *n*	30	30
Triglycerides, mmol/L	Mean ± SD	1.28 ± 0.7	2.5 ± 1.5^e^
	Range	0.4–3.9	0.6–7.8
	Total, *n*	42	42
eGFR, mL/min/1.73m^2^	Mean ± SD	73.4 ± 18.2	63.2 ± 16.8^e^
	Range	25–90	20–90
	Total, *n*	89	89
Transplant details			
Indication for transplant, *n* (%)	NAFLD	35 (35)	
	HCC^c^	13 (13)	
	ArLD	25 (25)	
	AIH	14 (14)	
	Haemochromatosis	2 (2)	
	Sarcoidosis	1 (1)	
	Other	10 (10)	
Child Pugh Score, *n (%)*	A	16 (17.8)	
	B	45 (50)	
	C	25 (27.8)	
	No cirrhosis	4 (4.4)	
UKELD	Mean ± SD	54.7 ± 5.1	
	Range	43–72	
	Total, *n*	100	
MELD	Mean ± SD	14.6 ± 5.5	
	Range	5–35	
	Total, *n*	93	
Post‐transplant immunotherapy regime^d^, *n* (%)	Prograf (Tacrolimus)	99 (99)	
	MMF	55 (55)	
	Prednisolone	98 (98)	
	Azathioprine	40 (40)	
Prograf (Tacrolimus) dosage, mg	Mean ± SD	7.2 ± 3.1	
	Mode	6	
	Range	2–16	
MMF dosage^d^, mg	Mean ± SD	1.9 ± 0.2	
	Mode	2	
	Range	1–2	
Prednisolone dosage^d^, mg	Mean ± SD	18.8 ± 3.1	
	Mode	20	
	Range	5–22.5	
Azathioprine dosage^d^, mg	Mean ± SD	105.6 ± 15.5	
	Mode	100	
	Range	75–125	

*Note:* Where applicable, where *n* does not equal 100, data were not available.

Abbreviations: AIH: autoimmune hepatitis; ArLD: alcohol‐related liver disease; CKD: chronic kidney disease; DBD: donation after brain death; DCD: donation after circulatory death; HCC: hepatocellular carcinoma; MELD: model for end‐stage liver disease; MF: mycophenolate mofetil; NAFLD: non‐alcoholic fatty liver disease; SD: standard deviation; UKELD: UK model for end‐stage liver disease.

^a^ 3‐months post‐op.

^b^ 1‐month post‐op.

^c^ As the primary indication for liver transplant.

^d^ Post‐operative regimen on initial discharge from hospital.

^e^ The difference between pre‐op and post‐op is significantly different using either the paired Student's *t*‐test or the Chi‐squared test.

**TABLE 3:** The change in total daily insulin requirements (in units) between time periods (pre‐, 1‐, 3‐ and 6‐most post‐transplant).

Time period (A)	Time period (B)	Mean difference (A‑B), units (%)	Std. error	Significance^b^	95% confidence interval for difference	
					Lower bound	Upper bound
1	2	9.4 (15.5)	3.8	*p* = 0.02	1.73	17.0
	3	17.1 (28.2)^a^	4.1	*p* < 0.0001	8.8	25.3
	4	26.9 (44.5)^a^	0.4	*p* < 0.0001	17.9	35.9
2	3	7.7 (15.0)^a^	2.0	*p* < 0.0005	3.6	11.7
	4	17.5 (34.3)^a^	3.0	*p* < 0.0001	11.5	23.5
3	4	9.9 (22.7)^a^	2.1	*p* < 0.0001	5.6	14.1

*Note:* Time periods: 1: Pre‐transplant. 2: 1‐month post‐transplant. 3: 3‐months post‐transplant. 4: 6‐months post‐transplant. Based on estimated marginal means.

^a^ The mean difference is statistically significant.

^b^ Adjustment for multiple comparisons: Bonferroni.

The difference in insulin requirements between pre‐ and at 6 months post‐transplant showed correlation with the use of tacrolimus as an immunosuppressant only. Spearman's Rank‐Order correlation was −0.21 for tacrolimus dosage; the higher the dose of tacrolimus, the lower the reduction in insulin requirement (*p* = 0.03). Spearman's Rank‐Order correlation for the other immunosuppressant dosages was: −0.03 (*p* > 0.05) for mycophenolate mofetil, 0.05 (*p* > 0.05) for prednisone, and − 0.003 (*p* > 0.05) for azathioprine. Although note that these were not independent contributions, and many patients were on multiple agents. A sample *T*‐test revealed there was no difference between patients on and off azathioprine or MMF treatment and the reduction in insulin requirement pre‐ and 6 months post‐transplant. This test was not done for patients on tacrolimus or prednisolone as almost all patients were on both immunosuppressants.

Table 4 shows the comparison of the mean difference of the insulin requirements before transplant and at 6 months after transplant between patients based on the indication for transplant. The greatest insulin reduction was observed for patients whose indication for transplant was ArLD (36.28 ± 9.9 units/day; 59% reduction) and NAFLD (32.4 ± 7.9 units/day; 45.5% reduction). Kruskal‑Wallis test neared significance but revealed no difference (*p* = 0.07) in the mean values between the different indications for transplant.

**TABLE 4:** The change in total daily insulin requirements (units) between pre‐transplant and 6 months post‐transplant.

Indication for transplant	Mean difference in insulin requirements, units (%)	Std. error	95% confidence interval for mean	
			Lower bound	Upper bound
NAFLD	32.4 (45.5)	7.9	16.4	48.4
HCC	4 (9.2)	7.2	−11.8	19.8
ArLD	36.3 (59)	9.9	15.8	56.7
AIH	16.1 (33.4)	7.1	0.69	31.6
Other	15 (29.9)	8.9	−5.2	35.2
All	26.9 (44.5)	0.4	17.9	35.9

Abbreviations: AIH: autoimmune hepatitis; ArLD: alcohol‐related liver disease; HCC: hepatocellular carcinoma; NAFLD: non‐alcoholic fatty liver disease.

Severity of liver disease had no impact on the mean difference in insulin requirements pre‐ and 6 months post‐transplant, in any of the scoring systems used. There was no difference in mean values of insulin requirement between Child‐Pugh scores using Kruskal Wallis test (*p* = 0.85). Similarly, there was no correlation between UKELD score or MELD and the mean difference in insulin requirement pre‐ and 6 months post‐transplant; Spearman's Rank‐Order Correlation 0.034 (*p* = 0.73) and 0.06 (*p* = 0.55), respectively.

### 3.3 Oral Anti‐Glycaemic Agents

Preoperatively, 54.3% of our population were receiving at least one oral anti‐glycaemic agent. This percentage was reduced to 25.6% at the first month after liver transplantation, 25.8% at the third month, and 27.9% at the sixth month post‐transplant. Cochran's Q test revealed that there was a statistically significant difference at least between two groups. Multiple post hoc comparisons, using the Bonferroni correction, showed that there was a statistically significant difference in the reduction of oral anti‐glycaemic agents from the first month (*p* < 0.0001) compared to pre‐transplantation. After transplant, there was not any statistically significant difference in the reduction of oral anti‐glycaemic agents between the first, third, and sixth months.

## 4 Discussion

This study has shown improved glycaemic control in patients with pre‐existing diabetes mellitus, illustrated by gradual reduction in insulin dose and oral anti‐glycaemic agent requirement in the months post‐transplant, with reduction in HbA1c. This suggests the detrimental effects of immunosuppressive therapy on insulin resistance and hyperglycaemic mechanisms are outweighed by the positive effects of restoration of hepatic glycaemic cellular function.

### 4.1 Restoration of Hepatic Glycaemic Metabolism and Hepatopancreatic Axis

The liver plays a central role in glucose regulation and lipid metabolism [18]. The accumulation of fat in the liver, a finding in liver disease such as NAFLD and ArLD, is linked to insulin resistance. The prevalence of a fatty liver on imaging amongst diabetic patients is between 50%–75% [19]. The same is true in reverse, with NAFLD being a predictor for future diabetes development [20]. Diabetes and glucose intolerance are highly prevalent in cirrhosis of any etiology, due to the effect of cirrhosis on insulin‐induced glucose uptake (insulin resistance) and pancreatic beta cell insulin secretion [21]. Profound peripheral insulin resistance is a feature of liver cirrhosis in both diabetic and non‐diabetic patients and is known to occur at an early stage of liver disease [21, 22]. Hyperinsulinaemia due to impaired hepatic degradation of insulin is a contributor [22, 23].

Hyperglycaemia and impaired glucose tolerance are also commonplace in patients with cirrhosis. This is thought to be multi‐factorial: reduced first pass metabolism of the liver of dietary glucose leading to higher levels of peripheral glucose, increased hepatic glucose production (reduced glycogen synthesis and gluconeogenesis) and reduced glucose oxidation [21, 22, 23]. Prolonged insulin resistance and hepatic induced hyperglycaemia lead to subsequent insulin hypersecretion from the pancreatic beta cells, leading to beta cell dysfunction, not capable of compensating for heightened levels of hyperglycaemia, indicative of diabetes [22]. This study suggests that by reintroducing a functioning liver, the normal regulation of hepatic glucose metabolism and hepato‐pancreatic axis is re‐established, leading to drastic improvements in diabetic treatment requirement and glycaemic control. This is supportive of previous research showing the improvement in glucose tolerance, insulin secretion, and sensitivity post‐liver transplant [22, 23].

### 4.2 Comparison With Previous Research

One case–control study found normalization of endogenous glucose production, improved insulin sensitivity, and remission of diabetes in 16/24 patients with pre‐existing diabetes 2 years post‐liver transplantation for HCC‐associated cirrhosis [22]. Another case–control study showed an improvement in oral glucose tolerance, fasting insulin levels, and insulin sensitivity post‐liver transplant for variable aetiologies [23].

There is limited research looking specifically at insulin requirements or HbA1c in those with pre‐existing diabetes post‐transplantation. Two single case reports describe remission of diabetes post‐liver transplant in patients with insulin‐dependent diabetes with normalization of HbA1c and discontinuation of insulin and oral anti‐glycaemic agents [3, 17]. Similarly, one small observational study showed reduction or cessation of insulin use in 5/9 patients with pre‐existing diabetes post‐transplant once corticosteroids stopped [10]. Another observational study showed a transient increase from 9/14 patients requiring insulin pre‐liver transplant to 13/14 at one year post‐transplant. This diminished at 3 years to 8/13 patients with a statistically significant reduction in total insulin requirements [11]. All studies attributed the improvement of diabetic control and/or remission of diabetes to the restoration of normal liver glucose metabolism post‐liver transplantation.

Interestingly, one case report of a patient with 10 years of insulin dependence achieved remission from diabetes post‐liver transplantation, but continued to have worsening of pre‐existing diabetic nephropathy and developed new diabetic retinopathy [17]. This suggests that despite improved glycaemic control, ongoing pathological complications of diabetes can continue.

These studies mostly show an initial transient increase in insulin requirement with immunosuppression that then reduces with improved glycaemic control after a prolonged period off immunosuppression. In addition, approximately 15% of patients post‐liver transplant develop new onset diabetes mellitus (variable diagnostic criteria, > 1‐month post‐surgery), with higher rates seen in males, ArLD, and tacrolimus therapy [24]. Conversely, despite a large majority of our cohort being male (69%), ArLD (28%) and having tacrolimus (97%), our data illustrate a rapid insulin requirement reduction even after 1 month. This is considerable, especially as immunosuppression with corticosteroids was still at high dosage (e.g., 20 mg prednisolone per day).

### 4.3 Effect of Liver Transplant on Oral Anti‐Glycaemic Agent Usage

We have shown a significant reduction in oral anti‐glycaemic agent use between pre‐ and 6 months post‐transplant. This likely reflects the same picture of re‐installation of functioning hepatic glucose metabolism and subsequent restoration of the hepato‐pancreatic axis. The biggest reduction in oral anti‐glycaemic agent use was between pre‐transplant (54.3%) and 1‐month post‐transplant (25.7%). This can be attributed to a concomitant insulin use in the weeks immediately following surgery (ongoing post‐surgical stress response and steroid use); there was an increase in total patients requiring insulin from 55.9% pre‐operatively to 78.2% at 1‐month post‐operatively. It is, however, noteworthy that insulin requirement within the subpopulation of patients on insulin pre‐transplant also reduced in this time.

In the pre‐transplant phase, in general, sulfonylureas are contraindicated due to end‐stage liver disease. Furthermore, many other oral agents are advised to be given with caution or not at all (metformin, sulphonyureas, sodium‐glucose transporter two inhibitors) [25]. Post‐transplant, with the restoration of normal liver function, these medications are no longer contraindicated, although many will be prescribed off‐license. This improved availability of more oral anti‐glycaemic medications could be a contributor to the reduction in insulin requirements post‐transplant.

### 4.4 Effect of Variables on Insulin Requirements

#### 4.4.1 Indications for Liver Transplant

Diabetes and glycaemic dysregulation are main contributors in the pathogenesis of NAFLD and are predictors of fibrosis progression in other aetiologies, including Hepatitis B and C viruses. Interestingly, patients with ArLD and NAFLD had the largest reduction in insulin requirements post‐transplant, although not statistically significant. This is despite continued hepatic insulin resistance contributed by ongoing subcutaneous and intraabdominal fat in patients with NAFLD and ArLD with metabolic overlap after liver transplantation [26]. Again, this highlights the importance of the liver in the role of insulin resistance and diabetes mellitus. Patients with AIH cirrhosis prior to transplant had a slightly lower reduction in insulin requirement, maybe due to the fact that they are placed on long‐term corticosteroids to prevent disease recurrence [27], whereas the other indications would have discontinued at 3 months as per protocol. The lowest recorded reductions in insulin post‐transplant were seen in patients with HCC. This may be explained by the fact these patients had less liver disease severity (less decompensation), as their primary indication was the cancer and not liver failure per se. It might be therefore that their insulin resistance was attributed less to the liver and more to peripheral insulin resistance in this sub‐group. It is noteworthy that the patients with both HCC and AIH as indications for transplant had lower starting doses of insulin than those with ArLD and NAFLD.

#### 4.4.2 BMI

Weight loss has long been linked to the improvement in glycaemic control and even reversal of type 2 diabetes [28]. Here we found a modest but significant reduction in BMI from 29.5 kg/m^2^ pre‐transplant to 27.9 kg/m^2^ post‐transplant, which may have influenced the improvement in glycaemic control and insulin requirement. This weight loss may only be transient, as previous research has shown that patients often gain excessive weight after liver transplantation [29]. Post‐surgical complications, hospital‐related weight loss, or intentional weight loss could be contributing factors to our findings, and we only collected data of BMI pre‐ and 6 months post‐transplant. Of note, the percentage of non‐smokers and non‐alcohol users increased significantly from 87.8% to 96.1% and 68.7% to 98.3% (*p* < 0.05), respectively. This could reflect lifestyle behavioral change leading to weight loss, improved glycaemic control, and reduced anti‐glycaemic agent requirement.

#### 4.4.3 Immunosuppression

In PTDM, CNI immunosuppressant agents causing insulin secretory dysfunction are attributed as the main risk factor for developing diabetes [12, 30]. In keeping with this, our study showed that a higher tacrolimus dose on initial discharge from hospital post‐liver transplant led to a lower reduction in insulin requirement between pre‐ and 6 months post‐transplant. Although we do not know what the dose of tacrolimus was beyond discharge.

At 1‐month post‐transplantation, our results already showed a non‐significant reduction in insulin requirement when patients were still on high‐dose steroids and with the possibility of post‐operative stress effect on glycaemic control. It is possible that the restorative effects of a new liver on glycaemic control may not have come into effect this early. There was then a significant reduction in insulin requirements at both 3‐ and 6‐months post‐surgery, when most patients would have stopped corticosteroids. The results epitomize the effect of the new liver on improved glycaemic control, which by the time this is likely to be fully operational.

We did not see any difference between the other immunosuppressant agent regimens (prednisolone, azathioprine or MMF) initiated post‐transplant on the reduction of insulin requirement. Note, nearly all were on corticosteroids (98.4%) and tacrolimus (97%)—with the only variation being between the choice of anti‐metabolite.

#### 4.4.4 Liver Disease Severity

One might speculate that the higher the severity of liver disease (and therefore poorer function), the greater the hepatogenous effect on dysglycaemia. However, there is limited evidence about the effect of severity scoring on glycaemic control post‐transplant in patients with pre‐existing diabetes. Our study did not find that the severity of liver failure using Child‐Pugh, UKELD, and MELD classification scoring prior to transplantation had an effect on the reduction in insulin requirement before and 6 months after transplantation. This is consistent with previous research that Child‐Pugh scoring has not significantly predicted the development of PTDM [11, 31].

#### 4.4.5 Reduction in Renal Function

There was a significant reduction in renal function from pre‐ to 6‐months post‐transplantation in the whole cohort (eGFR 75.4 to 54.6 mL/min/1.73m^2^) and the insulin sub‐population (eGFR 73.4 to 63.2 6 mL/min/1.73m^2^). Given that the kidneys play a key role in insulin clearance [32] and reduced renal function is known to cause reduced insulin requirements [33, 34], this reduction could be contributing to the reduction in insulin requirements. However, given that the eGFR was only reduced by 10 mL/min/1.73 mL/min/1.73m^2^ (14% reduction) between pre‐ and post‐transplant, the impact is likely to be small.

#### 4.4.6 Comparing Pre‐Existing Diabetes and PTDM

The majority of our patients had type 2 diabetes mellitus (91.5%). Many were insulin dependent prior to liver transplant, which is considered a sign of poorly controlled/advanced type 2 diabetes. It could be that in a patient group with heightened insulin resistance and glucose intolerance, the pathological impact of immunosuppression is offset by the benefits of a new functioning liver on glucose metabolism. Conversely, in patients without pre‐existing diabetes or defect in glucose regulation, there may be a profound perceived worsening of glycaemic control leading to PTDM. Alternatively, the underlying cause of the pre‐existing diabetes could be ‘hepatogenous’ in nature in these patients [22]. This would differentiate between pre‐existing hepatogenous diabetes and PTDM due to differing underlying pathophysiology and may help explain why pre‐existing diabetic control may improve whilst patients without diabetes prior to transplant may develop PTDM.

## 5 Strengths

The concept of looking at the effect of liver transplantation on glucose anti‐glycaemic agent (namely insulin) requirements and glycaemic control in patients with pre‐existing diabetes has not been reviewed on a large scale before, making the study concept novel. Even though the findings need to be validated by other transplant centers (with different ethnicity), the fact that this was undertaken in a large single center limits variations in clinical practice/drug prescribing and immunosuppression regimens.

## 6 Limitations

As an observational retrospective study, there are limits to the data available for collection. Our subgroup analysis of 100 patients on insulin prior to transplantation does not account for the further 43 patients on insulin prior to transplant who were excluded due to incomplete data. Although comparison of baseline characteristics between the included and excluded groups shows a similar reduction in the number of patients requiring insulin and markedly reduced HbA1c (Table S1).

Reviewing the complication rates of liver transplant was beyond the remit of this study and therefore we lacked data on the impact of early acute rejection (prevalence 20%) and rescue high dose pulsed corticosteroids (i.e., intravenous 1 g methylprednisolone for 72 h and prolonged 20 mg prednisolone therapy) on glycaemic control/insulin requirements. If anything, our findings may have been more pronounced if we had excluded patients with treated rejection from the analysis.

We did not look specifically at particular oral or other anti‐glycaemic agents; however, sulphonyureas and insulin are used most typically for diabetic control (> 90%), due to the tendency to primarily use these agents in patients with advanced or decompensated liver disease. To that end, we did not review the initiation of new post‐transplant anti‐glycaemic agents, such as metformin or Glucagon‐like peptide‐1 medications, which are contraindicated pre‐transplant.

We did not assess blood tacrolimus levels or doses of tacrolimus beyond initial discharge, as reducing doses/trough levels may well have been a contributor to further insulin requirement reduction. Finally, it should be noted that whilst the mean hemoglobin level did not fall between the pre‐ and post‐operative periods, anemia of any level can make HbA1c readings unreliable, and this should be considered when interpreting the reduction in HbA1c.

## 7 Future Research

More research needs to be performed to understand the pathogenesis behind our findings of improved glycaemic control represented by reduced insulin and oral anti‐glycaemic agent requirements post‐liver transplantation. Future research could focus on a multi‐centric European/continental pooled collective to validate the results in a well‐designed prospective study, with longer follow‐up to elucidate how glucose metabolism may change over time post‐liver transplantation.

## 8 Conclusion

Liver transplantation is a highly effective curative option for end‐stage liver disease and/or HCC, but our study also highlights that there are additional benefits in patients with pre‐existing type 2 diabetes, which in turn may have long‐term benefits on transplant survival and cardiovascular morbidity. We observed statistically significant reductions in diabetic treatment requirements (insulin and oral anti‐glycaemic agents) despite the burden of immunosuppressive drugs post transplantation.

## Author Contributions

W.H. was responsible for the concept of the project and lead. A.C. was lead in drafting the manuscript, data analysis, and helped collect data. C.A. was co‐lead in data analysis and helped with drafting the manuscript. O.A., N.S., L.K., A.N., A.P., S.U., A.A., and Z.K. contributed to the acquisition of data. S.M. contributed to project design and manuscript drafting/edits. F.B. helped with project design. M.A. provided expertise in hepatology, crucial for project design and manuscript critiquing. All authors helped with manuscript revision and final approval of the version to be published.

## Conflicts of Interest

The authors declare no conflicts of interest.

## Supporting information


**Table S1,** Description characteristics for population of those receiving insulin pre‐transplant comparing those included in the subpopulation analysis (*n* = 100) and those excluded (*n* = 43).

## Data Availability

The datasets generated during and/or analyzed during the current study are available from the corresponding author on reasonable request.
