# Supplementary material for: The Effect of Liver Transplantation on Anti‐Glycaemic Agents in Patients With Pre‐Existing Diabetes Mellitus: A Population‐Based Cohort Study
Source: J Diabetes. 2025 Jul 15;17(7):e70088. doi: 10.1111/1753-0407.70088 (PMC12263289; doi:10.1111/1753-0407.70088)
Supplement: Supplementary file 1 — Table S1, Description characteristics for population of those receiving insulin pre‐transplant comparing those included in the subpopulation analysis (n = 100) and those excluded (n = 43). [file JDB-17-e70088-s001.docx]

**Supplementary table 1:** Description characteristics for population of those receiving insulin pre-transplant comparing those included in the subpopulation analysis (n=100) and those excluded (n=43).

| **Characteristic** | | **Included** | | **Excluded** | |
| --- | --- | --- | --- | --- | --- |
|  | | **1** | **2** | **1** | **2** |
| *Demographics* | | | | | |
| Age, years | Mean ± SD | 55.8 ± 10.2 |  | 51.5 ± 10.6 |  |
|  | Range | 22-82 |  | 20-68 |  |
|  | Total, *n* | 99 |  | 43 |  |
| Sex, *n* (%) | Male | 63 (63) |  | 27 (63) |  |
|  | Female | 37 (37) |  | 16 (37) |  |
| *Diabetic history and control* | | | | | |
| Type of diabetes, *n* (%) | T2DM | 91 (91) |  | 35 (81.4) |  |
|  | T1DM | 9 (9) |  | 8 (18.6) |  |
| Duration of diabetes, months | Mean ± SD | 115.7 ± 87.1 |  | 78.5 ± 79.3 |  |
|  | Range | 12-384 |  | 12-276 |  |
|  | Total, *n* | 71 |  | 21 |  |
| On diabetic medication, *n* (%) | Insulin | 100 (100) | 84 (84) * | 43 (100) | 31 (91.1) |
|  | Oral anti-glycaemic agents | 22 (22) | 14 (14.1) | 6 (14) | 1 (2.6) |
|  | None | 0 (0) | 12 (12.1) * | 0 (0) | 3 (8.8) |
| HbA1c level, mmol/mol (%) | Mean ± SD | 55.7 ± 21.7  (7.2 ± 4.1) | 51.7 ± 18.8†  (6.9 ± 3.9) | 53.7 ± 14.1  (7.1 ± 3.4) | 43.2 ± 13.7†*  (6.1 ± 3.4) |
|  | Range | 20-138  (4-14.8) | 17-127  (3.7-13.8) | 28-85  (4.7-9.9) | 23-73  (4.3-8.8) |
|  | Total, *n* | 72 | 72 | 22 | 22 |
| Random glucose measurement, mmol/L | Mean ± SD | 9.8 ± 4.9 | 12.8 ± 6.6 ‡* | 10.9 ± 4.3 | 11.9 ± 4.9‡ |
|  | Range | 1.5-32.7 | 4-53 | 4.3-22.2 | 5.2-30.4 |
|  | Total, *n* | 88 | 88 | 31 | 31 |
| *Co-morbidities and associated laboratory values* | | | | | |
| Hb, g/dl | Mean ± SD | 113.1 ± 19.8 | 114.8 ± 17.7† | 108.4 ± 20.7 | 116.6 ± 16.8† |
|  | Range | 69-155 | 78-157 | 48-143 | 76-150 |
|  | Total, *n* | 67 | 67 | 22 | 22 |
| BMI, kg/m^2^ | Mean ± SD | 29.3 ± 5.4 | 27.1 ± 5.5 * | 28.3 ± 6.0 | 27.3 ± 5.8* |
|  | Range | 17.8-48.6 | 10.1-42.0 | 19-43.4 | 19.4-41.4 |
|  | Total, *n* | 92 | 92 | 36 | 36 |
| Triglycerides, mmol/L | Mean ± SD | 1.28 ± 0.7 | 2.5 ± 1.5 * | 2.0 ± 1.8 | 2.15 ± 0.8 |
|  | Range | 0.4-3.9 | 0.6-7.8 | 0.6-6.2 | 1.3-3.5 |
|  | Total, *n* | 42 | 42 | 10 | 10 |
| eGFR, mL/min/1.73m^2^ | Mean ± SD | 73.4 ± 18.2 | 63.2 ± 16.8 * | 78.3 ± 19.4 | 72.1 ± 23.8 |
|  | Range | 25-90 | 20-90 | 8-90 | 12-90 |
|  | Total, *n* | 89 | 89 | 36 | 36 |
| *Transplant details* | | | | | |
| Indication for transplant, *n* (%) | NAFLD | 35 (35) |  | 6 (14) |  |
|  | HCC § | 13 (13) |  | 2 (4.7) |  |
|  | ArLD | 25 (25) |  | 16 (37.2) |  |
|  | AIH | 14 (14) |  | 12 (27.9) |  |
|  | Haemo-chromatosis | 2 (2) |  | 0 (0) |  |
|  | Sarcoidosis | 1 (1) |  | 0 (0) |  |
|  | Other | 10 (10) |  | 7 (16.3) |  |
| Child Pugh Score, *n (%)* | A | 16 (17.8) |  | 5 (13.5) |  |
|  | B | 45 (50) |  | 25 (67.6) |  |
|  | C | 25 (27.8) |  | 5 (13.5) |  |
|  | No cirrhosis | 4 (4.4) |  | 2 (5.4) |  |
| UKELD | Mean ± SD | 54.7 ± 5.1 |  | 54.5 ± 4.9 |  |
|  | Range | 43-72 |  | 45-67 |  |
|  | Total, *n* | 100 |  | 43 |  |
| MELD | Mean ± SD | 14.6 ± 5.5 |  | 14.4 ± 6.3 |  |
|  | Range | 5-35 |  | 6-37 |  |
|  | Total, *n* | 93 |  | 42 |  |
| Post-transplant immunotherapy regime¶, *n* (%) | Prograf (Tacrolimus) | 99 (99) |  | 43 (100) |  |
|  | MMF | 55 (55) |  | 43 (100) |  |
|  | Prednisolone | 98 (98) |  | 23 (53.5) |  |
|  | Azathioprine | 40 (40) |  | 17 (38.5) |  |

1: Pre-op values. 2: 6-months post-op values.

† 3-months post-op. ‡1-month post-op. § As the primary indication for liver transplant. ¶ Post-op regimen on initial discharge from hospital.

* The difference between pre-op and post-op is significantly different using either paired Student’s t-test or Chi-squared test.

SD: Standard Deviation. CKD: Chronic Kidney disease. NAFLD: Non-Alcoholic Fatty Liver Disease. HCC: Hepatocellular Carcinoma. ArLD: Alcohol-related Liver Disease. AIH: Autoimmune Hepatitis. DCD: Donation after Circulatory Death. DBD: Donation after brain death. UKELD: UK model for End-Stage Liver Disease. MELD: Model for End-stage Liver Disease. MMF: Mycophenolate Mofetil.
